# Predicting failure of extubation and non-invasive respiratory support in critically ill patients: clinical complexity, limitations of traditional indices, and machine learning perspectives

**DOI:** 10.3389/fmed.2026.1791505

**Published:** 2026-05-20

**Authors:** Salvatore Notaro, Roberto Giurazza, Andrea Imparato, Fabrizio Falso, Salvatore Virno, Teresa Pasqualina Iovino, Marco Caiazzo, Ludovica Golino, Gianmarco Russo, Emilio Di Costanzo, Massimiliano Barberio, Marcello Sorrentino, Giuseppe Fiorentino, Giuseppe La Cerra, Clelia Esposito, Antonio Corcione, Antonio M. Esquinas, Eugenio Piscitelli

**Affiliations:** 1 Unit of Anesthesiology, Intensive Care and Postoperative Intensive Care, Department of Critical Care, AORN Ospedali dei Colli, Naples, Italy; 2 Department of Woman, Child, General and Specialized Surgery, University of Campania “Luigi Vanvitelli”, Naples, Italy; 3 Intensive Care Unit, Buon Consiglio Fatebenefratelli Hospital, Naples, Italy; 4 Unit of Respiratory Pathophysiology, AORN Ospedali dei Colli, Naples, Italy; 5 Unit of Interventional Pulmonology, AORN Ospedali dei Colli, Naples, Italy; 6 Intensive Care Unit, Hospital Universitario Morales Meseguer, University of Murcia, Murcia, Spain

**Keywords:** clinical decision support, extubation failure, intensive care, machine learning, non-invasive respiratory support, non-invasive ventilation, risk prediction

## Abstract

Failure of non-invasive respiratory support represents a major and unresolved clinical challenge in critically ill patients, both after extubation and during non-invasive ventilation or high-flow oxygen therapy used as primary treatment. Delayed or inappropriate escalation to invasive mechanical ventilation is associated with adverse outcomes, underscoring the need for reliable tools to timely identify patients at risk of failure. Traditional bedside indices provide simplicity and immediate applicability but show limited predictive accuracy and poor generalizability across heterogeneous clinical settings. In recent years, predictive models based on machine learning have been developed to improve risk stratification by integrating multiple clinical and physiological variables. Although early models demonstrated improved discrimination compared with conventional indices, their clinical impact has been constrained by retrospective designs, heterogeneous outcome definitions, limited external validation, and poor interpretability—limitations that collectively prevent meaningful cross-study comparisons and hinder safe clinical translation. More recent approaches have shifted toward dynamic and time-dependent models that incorporate early physiological trajectories and treatment response, offering predictions that are more consistent with real-world clinical decision-making processes. Nevertheless, high predictive accuracy alone does not ensure clinical usefulness in the absence of transparent decision support and integration into clinical workflows. Overall, current evidence suggests that predictive models should not replace clinical judgment but rather support timely, contextualized decisions by identifying modifiable risks. Future research should prioritize standardized outcome definitions, prospective multicenter validation, and the development of interpretable, workflow-integrated tools to enable safe and effective clinical translation.

## 1 Introduction

Failure of non-invasive respiratory support remains one of the most significant clinical challenges in the management of critically ill patients. Despite progress in mechanical ventilation (MV) strategies, the use of non-invasive ventilation (NIV), and high-flow oxygen therapy (HFOT) with high-flow nasal cannulas (HFNC), a significant proportion of patients experience respiratory deterioration, requiring intubation or early reintubation, with significant adverse consequences in terms of mortality, length of stay, and healthcare resource consumption (1, 2). Therefore, the ability to quickly recognize patients at risk of failure is still a fundamental objective of modern intensive care medicine.

The concept of “failure” in non-invasive respiratory support, however, is intrinsically heterogeneous. In the post-extubation setting, failure may include the need for reintubation, the use of NIV or HFNC as salvage therapy, or early death. Similarly, during NIV or HFNC as primary treatment, escalation to invasive ventilation represents a clinically relevant but defined endpoint with variable time windows and criteria across studies (1). This heterogeneity reflects the physiological and pathological complexity of the critically ill patient, in which respiratory, neurological, hemodynamic, and metabolic factors interact dynamically, making reliable prediction based on single parameters difficult.

The ability to correctly predict liberation from MV is a matter of high clinical relevance, since an unnecessary delay or premature liberation have been associated with adverse outcomes, prolonged MV and intensive care unit length of stay (3, 4). Indeed, an inappropriate untimely weaning from MV poses a significant strain on cardiovascular and respiratory system, as well as an inappropriate late weaning carries the risk of diaphragmatic atrophy, venous thromboembolism, ventilator-associated pneumonia and increased morbidity and mortality (4).

Traditionally, risk stratification of failure has been based on relatively simple clinical and physiological indices, such as the rapid shallow breathing index (RSBI), the ROX index, the HACOR score, or other composite scores derived from variables easily measurable at the patient’s bedside (4, 5). Although these tools have the advantage of simplicity and immediate clinical usefulness, numerous studies have demonstrated only moderate predictive performance and limited generalizability across different populations and settings (1, 6, 7). In particular, the use of static thresholds and point-in-time assessments does not capture the time-dependent nature of respiratory deterioration or the individual patient’s response to non-invasive support.

In recent years, interest within predictive models based on machine learning (ML) has grown exponentially, powered by the availability of large clinical databases and the ability of these algorithms to integrate a wide number of variables simultaneously. The first ML models applied to predict extubation or NIV failure showed superior discrimination in comparison with conventional scores, suggesting that multivariate approaches could grasp intricate clinical patterns not evident with standard techniques (6). However, initial interest was progressively tempered by the emergence of substantial methodological limitations, including retrospective designs, non-robust internal validation, heterogeneous outcome definitions, and poor transferability of models throughout different clinical contexts (8).

Although often underestimated, the clinician should always consider the gap between statistical significance and clinical utility. Indeed, a high area under the receiver operating characteristic (ROC) curve does not necessarily guarantee improved bedside decision-making, especially when the model does not provide guidance on the optimal timing of intervention or is not integrated into clinical processes (9–11). In this sense, numerous ML models have proven to be ex post classification tools, rather than true prospective decision-making aids, limiting their impact on daily clinical practice.

More recently, some studies have begun to examine alternative approaches, focusing on the analysis of physiological trajectories and early changes in respiratory parameters in the first hours of non-invasive support. This paradigm shift, which focuses on dynamic trends over isolated baseline values, has demonstrated encouraging results, especially when combined with external testing processes on independent, multicenter cohorts (12). This evidence suggests the additional value of ML relies on its capacity to incorporate the patient’s initial clinical response and adapt to the practical clinical decision-making context.

In parallel, a wider interest in the function of artificial intelligence (AI) in clinical decision-making has developed. It is clear that prediction algorithms, although sophisticated, cannot replace clinical judgment, but should rather serve as early warning systems or decision support systems, helping to reduce uncertainty in clinical decisions, without introducing dangerous automatisms (9, 10). In the context of non-invasive respiratory support, this matter is of utmost importance, as late or inappropriate intubation decisions can have significant clinical consequences (13).

Taking these considerations into account, there is a need for a critical synthesis that integrates consolidated clinical evidence with the most recent developments in predictive models, clarifying what has actually been learned so far and identifying the most promising future directions. The purpose of this review is to critically examine the clinical complexity of non-invasive respiratory support failure, discuss the constraints of established indices and early ML approaches, and identify the conditions under which prediction algorithms can realistically contribute to improving the management of critically ill patients (14).

## 2 Rationale and literature selection

This review was structured as a narrative, yet comprehensive, overview of the current literature. The conceptual framework guiding our literature selection focused on three main pillars: the pathophysiological complexity of non-invasive respiratory support and extubation, the established limitations of traditional bedside indices, and the translation of ML algorithms into clinical decision support. To capture both foundational knowledge and contemporary advancements, literature searches were conducted in major databases (PubMed/MEDLINE, Scopus). For traditional clinical indices, we included pivotal historical studies and widely cited validation cohorts, regardless of publication year. Conversely, for the sections discussing artificial intelligence and dynamic modeling, we prioritized peer-reviewed articles, multicenter validations, and critical care guidelines published predominantly within the last decade. We also manually retrieved relevant pivotal articles from the reference lists of included studies and systematic reviews. Articles focusing on purely technical AI architectures, without clear clinical correlations, were excluded to maintain a strong bedside-oriented perspective.

## 3 Clinical complexity of extubation failure and non-invasive respiratory support

A significant challenge in managing non-invasive respiratory support is the heterogeneous definition of failure. Indeed, in literature, post-extubation failure encompasses early reintubation, the need for rescue NIV or HFNC, or mortality within varying timeframes (1, 15). This heterogeneity reflects not only variations in methodology between studies, but also different clinical approaches to post-extubation management, limiting both the comparability of results and the generalizability of prediction algorithms (16). Similarly, in the context of NIV or HFNC as primary treatment, failure is rarely a sudden event, but it often presents as a progressive loss of efficacy of the ventilatory support (12, 17). In these patients, the decision to intubate is strongly influenced by dynamic factors such as the initial treatment response, interface tolerance, gas exchange trends, and the appearance of signs of respiratory fatigue or hemodynamic instability (6, 18). Consequently, failure of non-invasive respiratory support cannot be interpreted as a single event, but it represents the unfavorable outcome of an intricate interplay among respiratory, neurological, cardiovascular, and systemic components, as well as the duration of previous mechanical ventilation and the presence of significant comorbidities. (1, 9, 19). Particularly, level of consciousness and airway protection emerge as central determinants, stressing the key role of the embedding between neurological control and respiratory function (7, 13). Additionally, frequently underrecognized conditions, such as intensive care-acquired muscle weakness or cardiopulmonary congestion, may considerably contribute to post-extubation clinical deterioration (20, 21).

Pathophysiological mechanisms also vary by clinical phenotype. In hypoxemic patients, failure often stems from disease progression, worsening ventilation-perfusion mismatch, or increased work of breathing (22, 23). Conversely, in hypercapnic patients, alterations in respiratory drive, muscle fatigue, and patient-ventilator asynchrony are the primary drivers of NIV intolerance (24, 25).

Finally, failure of non-invasive respiratory support is also influenced by the clinical context: intubation thresholds, team expertise, and resource availability introduce a degree of decisional variability that purely statistical models struggle to capture (10, 11).

Considering these premises, failure of non-invasive respiratory support must be considered an evolving and multidimensional phenomenon, dependent on the interaction between patient pathophysiology, response to treatment, and clinical decision-making (1, 9). Understanding this complexity is essential for critically evaluating the performance of risk stratification tools and for correctly interpreting the potential contribution of advanced prediction algorithms.

To bridge the gap between theoretical potential and reliable ML applications, the heterogeneous concept of clinical failure must be translated into standardized, operational definitions. Since ML algorithms require unambiguous target labels for training, future ML development should adopt a structured, multi-layered approach to define clinical failure.

To ensure reproducibility, algorithms must rely on unequivocal events, such as the requirement for endotracheal intubation or all-cause mortality within a strictly defined timeframes (e.g., 48–72 h post-extubation or following the initiation of support). However, since clinical deterioration is a continuum, models should also be trained to forecast intermediate failure states, including escalation of respiratory support (e.g., transitioning from HFNC to NIV) or crossing of predefined critical physiological thresholds (e.g., refractory hypoxemia or worsening respiratory acidosis). Furthermore, rather than classifying failure as a simple static dichotomous outcome, future research should leverage survival analysis frameworks. Predicting the specific time to intubation aligns more closely with the continuous monitoring environment of the intensive care unit.

By sorting these operational definitions, future ML models can provide clearer, more actionable predictions that directly support clinical decision-making.

## 4 Challenges of typical clinical indices in predicting failure

Over the past few decades, numerous clinical indices have been proposed to support risk stratification for extubation and non-invasive respiratory support failure. Among these, the most widely used are the rapid shallow breathing index (RSBI), the ROX index, the HACOR score, based on respiratory and hemodynamic parameters easily measurable at the patient’s bedside.

RSBI, first described by Yand and Tobin in 1991, is the ratio between respiratory rate (RR) and tidal volume (Tv) and expressed as breaths/min/L (26); a value greater than 105 breaths/min/L has been identified as predictive of unsuccessful weaning (27). The ROX index, defined as the ratio of oxygen saturation measured by pulse oximetry and fraction of inspired oxygen (FiO2) to RR, was first described by Roca et al. in 2019 to predict the outcome of HFNC in patients with acute hypoxemic respiratory failure (28). In their paper, the authors concluded that a ROX index ≥4.88 measured at 2, 6, or 12 h after HFNC initiation was associated with a lower risk for intubation, whereas a ROX index of <3.85 was associated with a high risk of failure, leaving a grey zone between 4.88 and 3.85 (28). One meta-analysis suggested that ROX index could be used to discriminate patients at risk of HFNC failure in acute hypoxemic respiratory failure associated with COVID-19 (29). The HACOR score is based on different clinical variables, including heart rate, acidosis, consciousness, oxygenation and respiratory rate; it was first introduced by Duan and colleagues in 2017 to predict NIV failure in hypoxemic patients, with a score greater than 5 being a predictor of NIV failure (30) (Table 1). The same results were also confirmed in chronic obstructive pulmonary disease (COPD) patients (31). Both in hypoxemic and hypercapnic patients with HACOR score >5, early intubation was associated with reduced mortality (30, 31). RSBI, ROX index and HACOR score are summarized in Table 2.

**Table 1:** HACOR score.

Variable	Value	Score
Heart rate	≤ 120	0
	> 120	1
Acidosis	≥ 7.35	0
	7.30–7.34	2
	7.25–7.29	3
	< 7.25	4
Consciousness (GCS)	15	0
	13–14	2
	11–12	5
	≤ 10	10
Oxygenation (PaO₂/FiO₂ ratio)	> 200	0
	176–200	2
	151–175	3
	126–150	4
	101–125	5
	≤ 100	6
Respiratory rate	≤ 30	0
	31–35	1
	36–40	2
	41–45	3
	> 45	4

**Table 2:** Typical clinical indices analyzing failure of non-invasive respiratory support.

Clinical index	Analyzed variables/calculation	Setting	Meaning
Rapid shallow breathing index (RSBI)	$\frac{\text{Respiratory rate}}{\text{Tidal volume}}$	Intubated and ventilated patients	Values >105 breaths/min/L are associated with unsuccessful weaning from intubation
ROX index	$\frac{\mathrm{SpO}2/\mathrm{FiO}2}{\text{respiratory rate}}$	Patients in acute hypoxemic respiratory failure receiving HFNC oxygen therapy	<3.85: high risk of failure 3.85–4.88: grey zone >4.88: low risk of failure
HACOR score	Sum of points assigned to: heart rate, acidosis, consciousness, oxygenation and respiratory rate (see Table 1)	Patients in acute (both hypoxemic and hypercapnic) respiratory failure receiving NIV	>5: predictor of NIV failure

Despite their broad use in clinical practice, these tools have intrinsic limitations that hinder their accuracy as true decision aids (1, 9). A first fundamental limitation concerns the static nature of most traditional indices that are point-assessments performed at specific time points, such as immediately before extubation or in the first hours of NIV or HFNC support. However, because respiratory decline is a progressive process, isolated measurements fail to capture the temporal trajectories of neurological status or gas exchange that often precede clinical collapse (6, 13). The inability to capture such temporal trajectories intrinsically limits the predictive value of isolated measurements.

A second major constraint is the reduction of pathophysiological complexity to a limited number of variables. While this simplification facilitates immediate application, it entails a significant loss of clinically relevant information. As already stated, the failure of non-invasive respiratory support results from the interaction of multiple systems—respiratory, cardiovascular, neurological, and metabolic—which cannot be represented by one or two dominant parameters (1, 21). Consequently, patients with similar values for the same index may follow profoundly different clinical trajectories.

The heterogeneity of the populations for development and validation constitutes a further key element. Many clinical indices have been developed in selected cohorts, often single center, with restrictive inclusion criteria and specific care settings. When applied to different populations—for example, hypoxemic versus hypercapnic patients, or settings with different intubation decision thresholds—their performance tends to decline significantly (15, 16). This limited generalizability compromises their use as universal decision-making tools.

Another weakness is the use of rigid cut-offs for risk classification. Binary risk classification ignores inter-individual variability. Strict thresholds can lead to either premature intubation or dangerous delays in escalating care (6, 17).

It should also be considered that most traditional indices have been evaluated primarily in terms of statistical accuracy, utilizing measures like sensitivity, specificity, or area under the ROC curve (27, 29, 30). Although useful for methodological comparisons, these metrics do not guarantee improved clinical decision-making or a direct effect on patient outcomes (9, 10). In the absence of structured embedding within clinical processes, these predictive tools risk remaining descriptive rather than truly transformative.

Finally, many traditional clinical indices do not explicitly consider the decision-making component that influences the failure of non-invasive respiratory support, such as intubation threshold, team experience and resource accessibility (9, 11).

Taken together, these limitations explain why, despite the widespread availability of risk stratification tools, the prediction of extubation failure and non-invasive respiratory support remains imprecise and poorly reliable in daily practice (1, 9). These crucial challenges have created the basis for the development of alternative approaches, oriented towards multivariate and dynamic models, with the aim of overcoming the intrinsic restrictions of classic clinical indices.

## 5 From clinical models to early machine learning approaches: promises and early obstacles

Recognition of the intrinsic shortcomings of standard clinical indices has spurred, in recent years, growing interest in ML-based predictive approaches applied to non-invasive respiratory support and the post-extubation period. These models seek to overcome the univariate simplification of traditional scores by simultaneously integrating a large number of clinical, physiological, and laboratory variables, with the aim of embracing intricate patterns associated with the risk of failure (6, 19).

Early studies in this area have demonstrated that supervised algorithms, such as random forests, support vector machines, and K nearest neighbors (K-NN) algorithm, can attain better discriminative effectiveness compared to conventional bedside indices in predicting reintubation or NIV failure (6, 18). In particular, the combination of different variables—including vital signs, blood gas analysis data, and demographic information—has allowed the modeling of nonlinear relationships that are difficult to capture with standard techniques (19).

Zhao et al. developed a ML model called Categorical Boosting (CatBoost), based on 89 clinical and laboratory variables for predicting extubation failure in critically ill patients, using data from 16,189 patients from the Medical Information Mart for Intensive Care (MIMIC)-IV database; the model was also prospectively validated in cardiac intensive care unit (ICU) patients to ensure reliability. Using the recursive feature elimination (RFE) algorithm, 19 key features were selected, including age, body mass index, stroke, heart rate, respiratory rate, mean arterial pressure, peripheral oxygen saturation, temperature, pH, central venous pressure, tidal volume, positive end-expiratory pressure, mean airway pressure, pressure support ventilation (PSV) level, mechanical ventilation (MV) durations, spontaneous breathing trial success times, urine output, crystalloid amount, and antibiotic types. The two most influential variables were the duration of mechanical ventilation and the level of Pressure Support (PSV) required prior to extubation. After hyperparameter optimization, the CatBoost model achieved an AUROC of 0.835, significantly outperforming traditional weaning indices (like the RSBI) and other standard scoring systems (6).

Similarly, Zhang et al. introduced a novel real-time machine-learning framework designed to forecast the need for invasive mechanical ventilation (IMV) by leveraging only non-invasive clinical data. Unlike conventional methods that rely on invasive arterial sampling, this real-time warning system was optimized for pre-hospital care and disaster medicine. Body Mass Index (BMI), neurological status (Glasgow Coma Scale), and patient age were identified as the primary factors influencing the prediction of NIV failure. The model highlighted how the multivariate integration of clinical and laboratory variables allows more accurate risk stratification than standard techniques. Indeed, it achieved an AUC value of 0.935 and 0.727, in the internal validation and in the multi-center validation using the AmsterdamUMCdb database, respectively. These values were greater than those of other traditional risk adjustment algorithms, such as P/F ratio and oxygenation saturation index (OSI) (32).

However, the initial interest was quickly accompanied by the emergence of substantial methodological issues. In particular, several authors pointed out the limitations of models based on static patient assessments. A systematic review by Gerry et al. critically appraises early warning scores and highlights substantial methodological weaknesses, including static modeling and insufficient adaptation to evolving clinical status. The authors also note that many early warning scores rely on static predictors and do not adequately account for changes in patient condition or treatment effects, leading to potential miscalibration and risk of false predictive confidence as clinical scenarios change (33).

One of the main limitations concerns the predominantly retrospective and observational design of many studies, often based on single-center databases or administrative registries. Typically, these studies employ a split-sample approach, using internal training and test cohorts to develop and initially verify the algorithms within a single-center database. However, this approach exposes models to selection bias and limits the possibility of robust prospective validation, reducing their transferability to everyday clinical practice (9, 10). To be safely transferred to clinical practice and ensure generalizability across different populations, models require rigorous external validation, i.e., testing the algorithms on independent datasets from different institutions. While some high-performing models have demonstrated robustness through external cohorts (32), the overall lack of external validation and the absence of prospective testing remain the major obstacles. Prospective validation in real-world clinical environments is the final necessary step to confirm that ML-driven models actually improve decision-making and patient outcomes without unintended biases.

Another major limitation is the heterogeneity of the outcomes used to train and evaluate the models. As previously highlighted, failure of non-invasive respiratory support is defined in various ways across studies, including different endpoints such as reintubation, therapeutic escalation, or early mortality. This variation compromises direct comparisons between models and hinders the construction of generalizable forecasting instruments (1, 16).

Many high-performance algorithms function as “black boxes,” providing a probability of failure without clarifying the relative contribution of individual variables. In the intensive care setting, where clinical decisions have immediate and potentially irreversible consequences, this indistinctness can reduce clinician confidence and limit their adoption (9). Consequently, forecasting precision, even if high, does not automatically translate into clinical utility. Therefore, although several ML models have demonstrated improved discriminatory performance compared with traditional clinical indices, their adoption in clinical practice has been limited by poor interpretability and the perception of black-box decision-making. In this context, recent studies have suggested that the integration of explainable artificial intelligence (XAI) techniques, such as SHAP (SHapley Additive exPlanations) or LIME (Local Interpretable Model-agnostic Explanations), may enhance clinician trust by clarifying feature relevance in intubation prediction models, without necessarily compromising predictive performance (34, 35).

A frequently overlooked aspect in early studies also concerns the timing of prediction. Many models were developed using data collected close to the failure event, when clinical deterioration is already underway (23). Although such models can identify high-risk patients, their value as prevention tools is limited, as they do not offer sufficient time to modify the therapeutic strategy (6, 12). This draws attention to the substantial difference between “diagnostic” prediction and truly anticipatory decision support.

An additional crucial issue is the phenomenon of dataset shift and the lack of adequate external validation. Models developed using specific populations can lose performance when applied to different contexts, characterized by different clinical practices, available resources, or patient profiles. The paucity of multicenter studies and independent external validation remains one of the main obstacles to the clinical translation of ML models (10, 17).

Finally, it should be emphasized that many early ML approaches have focused primarily on maximizing optimization of accuracy measures, without systematically assessing their impact on clinical decision-making and clinical outcomes. In the absence of decision curve analyses or pragmatic implementation studies, these models risk remaining academic tools, with limited impact on daily practice (9, 12).

Overall, early ML approaches have displayed marked potential for improving the prediction of non-invasive respiratory support failure in comparison with traditional indices. However, methodological weaknesses, outcome heterogeneity, poor interpretability, and the lack of prospective validation have pointed out the need for conceptual and methodological evolution. These considerations have established the basis for the creation of additional mature models, oriented not only at predictive performance but also at embedding within the clinical decision-making process.

## 6 Dynamic approaches and time-dependent models: toward clinically relevant prediction

The limitations that emerged in early, static ML approaches have progressively driven research toward dynamic and time-dependent models, designed to integrate not only baseline values but also the longitudinal progression of clinical parameters during non-invasive respiratory support, adhering more closely with clinical reality where the risk of failure is not static, but it changes in response to therapy and disease progression (12, 17). Recent studies have demonstrated that analyzing temporal trajectories—such as changes in respiratory rate, PaO₂/FiO₂ ratio, or pressure support over time—provides superior prognostic information compared to isolated snapshots (22, 23). In particular, early failure to respond to non-invasive support appears as a key signal of impending failure, suggesting that the predictive value lies in the dynamics of the response rather than the patient’s initial state (12). A comparison between static and dynamic models is reported in Table 3.

**Table 3:** Comparison between static and dynamic predictive models in non-invasive respiratory support.

Feature	Static models	Dynamic models
Data input	Single time-point (baseline)	Repeated, time-updated data
Temporal resolution	Fixed	Continuous or sequential
Clinical interpretation	Snapshot of patient status	Evolution of patient trajectory
Ability to capture treatment response	Limited	High
Prediction update	Not updated after initial calculation	Continuously updated
Clinical utility	Risk stratification	Real-time decision support
Examples	RSBI, ROX, HACOR	Time-series ML models, dynamic risk scores

In support of this approach, Pan et al. demonstrated that monitoring these trends in the early hours of non-invasive support allows for prompt detection of deterioration patterns (22). Consistently, Fu et al. highlighted that models able of iteratively updating risk estimates during treatment can identify clinical deterioration before overt clinical signs appear, offering a more precise tool for managing patients with uncertain initial responses. This dynamic approach appears particularly relevant in non-invasive respiratory support, where the timing of intubation is a critical element of outcome (18).

Yu et al. have emphasized that the combination of time-dependent variables not only enhances the statistical performance of models but it also provides more clinically useful information for decision-making, allowing the identification of time windows in which therapeutic escalation may still be modifiable and clinically beneficial (12).

By explicitly considering how a patient reacts to NIV or HFNC within the first few hours, these models can differentiate between high-severity patients who respond well to treatment and seemingly stable patients who are actually deteriorating. This allows clinicians to identify specific “time windows” where therapeutic escalation remains modifiable and potentially beneficial (12, 17).

In spite of these advances, dynamic models also show substantial methodological challenges. Managing time-series requires highly detailed and high quality data, and this cannot be always guaranteed in actual clinical environments or in units with limited resources (10).

Another limitation concerns the explainability of time-dependent models. Although they deliver a more exact view of the dynamic pathophysiology of the critically ill patient, the increase of the algorithm complexity can accentuate the problem of decision-making transparency (9).

It is also important to highlight that, in most available studies, the evaluation of dynamic models is still predominantly based on statistical assessment measures, while evidence on their prospective impact on clinical results continue to be limited. The lack of pragmatic studies comparing decision-making strategies supported by time-dependent models with standard practice constitutes a major gap in the current literature (12, 17).

In conclusion, while the transition to time-dependent modeling is a crucial step toward precision medicine in the ICU, its true utility will depend on the successful integration of forecasting accuracy with interpretability and bedside feasibility.

## 7 Clinical implementation, interpretability, and incorporation within clinical decision processes

Translating ML models from research settings to daily clinical practice represents one of the main current challenges in non-invasive respiratory support. Despite the methodological advances of ML approaches, their effective clinical implementation requires that prediction algorithms are not only merely accurate, but also interpretable, reliable, and seamlessly integrable within current clinical workflows (9, 10).

The clinical integration of ML models is primarily hindered by limited interpretability. Indeed, the “black box” nature of high-performance algorithms often obscures the weight of individual variables, undermining clinician trust and leading to rejection of these tools in favor of traditional and explainable clinical judgement. To tackle this critical issue, interest in XAI approaches has grown, aiming to make the contribution of individual variables to the final prediction more transparent. Methods for local and global explainability allow clinicians to visualize the parameters driving risk within specific time windows, providing a clearer predictive rationale (6, 18). It must be emphasized, however, that high predictive accuracy—as measured by AUC or sensitivity/specificity—is necessary but not sufficient for clinical usefulness: a model that performs well statistically but lacks transparency, timely integration into workflows, and actionable output will have limited impact on bedside decision-making. In clinical practice, XAI can be incorporated into electronic health records (EHR) through visual dashboards. For instance, using tools like SHAP values, a model can provide real-time, patient-specific information, showing exactly which parameters (such as a declining PF ratio or an increasing RR, are driving the prediction of impending NIV failure.

However, XAI presents some limitations that must be considered before widespread adoption. First, these methods illustrate statistical associations rather than true pathophysiological causality. Second, XAI adds extra complexity in high-pressure critical care environments, risking cognitive overload and alert fatigue. Lastly, they require adequate training of clinical staff to avoid misreading’s, oversimplifications and automation bias, where the clinicians may place excessive trust in the model, potentially ignoring their direct clinical assessment of the patient.

An additional critical element is embedding within clinical procedures. Forecasting models that operate as isolated tools, not integrated into hospital information systems or decision-making protocols, tend to have a limited impact on daily practice (10). Conversely, early warning systems embedded in electronic health records, capable of providing dynamic risk updates and contextualized suggestions, are more likely to support timely and consistent decisions (12). Furthermore, the clinical utility of a model depends heavily on the timing of the prediction. Estimates provided too early may be inaccurate, while those provided too late offer little room for intervention. The true value of these algorithms lies in identifying the optimal window for therapeutic escalation (12, 17).

An often-overlooked aspect concerns the impact of prediction algorithms on clinicians’ behavior. Decision-support systems can shift intubation thresholds and modify monitoring strategies, yet the lack of prospective studies assessing these behavioral effects makes the actual impact on clinical outcomes unclear (9, 10).

Finally, the sustainability and continuous updating of models represent a further challenge. Changes in clinical practices, patient populations, or respiratory support technologies can lead to a progressive deterioration of predictive performance, a phenomenon known as “model drift.” The need to monitor and periodically update models requires dedicated infrastructure and clear governance, aspects that are not always available in actual clinical practice (10).

In conclusion, successful clinical implementation requires a delicate equilibrium between accuracy, interpretability, and functional integration. Moving from purely forecasting models to true decision-support tools is essential for transforming the potential of machine learning into concrete clinical benefits at the bedside.

## 8 Discussion

The prediction of extubation failure and non-invasive respiratory support is an area in which technological progress has, at least in part, outpaced the ability of clinical practice to systematically integrate it. The evidence discussed in this review clearly shows that the problem does not lie in the lack of prediction algorithms, but rather in the difficulty of translating such models into clinically useful, reliable, and contextualized tools (1, 9).

### 8.1 Controversies and schools of thought

One of the main controversies concerns the optimal timing of intubation in patients treated with non-invasive respiratory support. On one hand, some authors argue that early identification of the risk of failure should promote timely escalation to invasive ventilation, in order to avoid the clinical deterioration associated with late intubations (13). On the other hand, there is a concern that overly sensitive prediction algorithms may induce premature intubations, exposing patients to avoidable risks associated with invasive MV (1).

A second area of debate concerns the comparison between bedside clinical indices and ML models. While traditional scores offer simplicity and transparency, they are often inadequate to represent the complexity and dynamics of critically ill patients. In contrast, ML models show greater discriminatory capacity although clinical integration limits their routine adoption (6, 9).

### 8.2 Methodological gaps and limitations of the current literature

Despite the growing number of studies, substantial methodological shortcomings continue. First, heterogeneity in the definition of failure outcomes makes comparisons between models difficult and hinders evidence synthesis (1, 16). Second, most models were developed in retrospective settings and validated internally, with a significant lack of multicenter external validation (10).

A further gap concerns the limited prospective evaluation of the models’ clinical impact. Most studies focus on statistical assessment measures, while rare studies analyze how the use of a predictive model concretely modifies the decision-making process, the timing of intubation, or clinical results (12). This limitation is particularly relevant in a context where clinical decisions are strongly influenced by team experience and organizational factors.

### 8.3 From prediction to decision support

A key message emerging from this review is the need to shift the focus from risk prediction to clinical decision support. Models that provide a static probability of failure, without indicating the optimal timing of intervention or the underlying pathophysiological rationale, offer limited value in daily practice (9). Conversely, dynamic and time-dependent approaches, capable of updating risk based on treatment response, appear more consistent with the clinician’s actual decision-making process (12, 17).

In this context, the combination of explainable AI tools may represent a key stage in increasing clinician confidence and supporting active use of models to facilitate shared and contextualized decisions.

### 8.4 Clinical consequences and upcoming developments directions

Considering the available evidence, the future of prediction algorithms in non-invasive respiratory support will rely on the capacity to address three main challenges: standardization, prospective validation, and clinical integration. The shared definition of failure endpoints, the adoption of multicenter designs, and the assessment of decisional impact are essential prerequisites for true clinical translation (1, 10).

Furthermore, it is likely that the most useful models will not be those with the highest forecasting precision, but those capable of identifying time windows of modifiable risk, supporting the clinician in weighing early escalation and a conservative approach. In this sense, models should be conceived as warning and support tools, rather than as independent decision-making systems. Explainability should therefore not be considered an ancillary feature, but a prerequisite for the responsible deployment of machine learning models in high-risk clinical environments, where transparency and accountability are essential for adoption, clinical trust, and patient safety (6, 36).
